# Genetic Divergence Disclosing a Rapid Prehistorical Dispersion of Native Americans in Central and South America

**DOI:** 10.1371/journal.pone.0044788

**Published:** 2012-09-06

**Authors:** Yungang He, Wei R. Wang, Ran Li, Sijia Wang, Li Jin

**Affiliations:** 1 Chinese Academy of Sciences and Max Planck Society (CAS-MPG) Partner Institute for Computational Biology, Shanghai Institutes for Biological Sciences, Chinese Academy of Sciences, Shanghai, China; 2 Key Laboratory of Computational Biology, CAS-MPG Partner Institute for Computational Biology, Chinese Academy of Sciences, Shanghai, China; 3 FAS Center for Systems Biology, Harvard University, Cambridge, Massachusetts, United States of America; 4 Ministry of Education Key Laboratory of Contemporary Anthropology, School of Life Sciences and Institute of Biomedical Sciences, Fudan University, Shanghai, China; Aarhus University, Denmark

## Abstract

An accurate estimate of the divergence time between Native Americans is important for understanding the initial entry and early dispersion of human beings in the New World. Current methods for estimating the genetic divergence time of populations could seriously depart from a linear relationship with the true divergence for multiple populations of a different population size and significant population expansion. Here, to address this problem, we propose a novel measure to estimate the genetic divergence time of populations. Computer simulation revealed that the new measure maintained an excellent linear correlation with the population divergence time in complicated multi-population scenarios with population expansion. Utilizing the new measure and microsatellite data of 21 Native American populations, we investigated the genetic divergences of the Native American populations. The results indicated that genetic divergences between North American populations are greater than that between Central and South American populations. None of the divergences, however, were large enough to constitute convincing evidence supporting the two-wave or multi-wave migration model for the initial entry of human beings into America. The genetic affinity of the Native American populations was further explored using Neighbor-Net and the genetic divergences suggested that these populations could be categorized into four genetic groups living in four different ecologic zones. The divergence of the population groups suggests that the early dispersion of human beings in America was a multi-step procedure. Further, the divergences suggest the rapid dispersion of Native Americans in Central and South Americas after a long standstill period in North America.

## Introduction

The genetic history of human populations, especially their colonization and migration, contains essential information for understanding the genetic structure of human populations as well as the genome landscape of human individuals. America is the continent most recently colonized by human beings. While mysteries about colonization in Eurasia and migration in Africa were partially clarified by genetic studies [1]–[4], many questions persist about the genetic history of Native Americans, and this remains one of the last few frontiers in the study of genetic history of our species [5], [6].

Migration models regarding the initial entry into America is one of the essential topics in genetic studies of Native Americans. Different migration models were proposed over the past few decades for the peopling of America when both archaeologic and genetic evidence suggested that populations related to Asians were the first successful colonizers to reach America during the last glacial maximum around 20 thousand years (kyr) before the present (BP) or even earlier [7], [8]. Many archaeologic sites reveal the presence of humans before 13 kyr BP, such as Schaefer and Hebior in Wisconsin, La Sena in Nebraska, and Lovewell in Kansas [7]. The dominance of the Clovis culture (about 13 kyr BP) in sites across North America was believed to be strong evidence for a single-wave migration in the pre-Clovis period [9]. Genetic studies largely support the model of single-wave migration based on mitochondrial DNA (mtDNA) and Y chromosome data [8], [10]–[13]. Many other models of more complicated migration scenarios have also been proposed, although some of them have been largely rejected by recent studies. For example, a tripartite model was proposed based on linguistic, archaeologic, and dental evidence in the 1980s [14]; a recent genetic study of mtDNA introduced a three-stage colonization model and the recent three-stage model has little similarity to the previous tripartite model [15], [16]. Furthermore, using short tandem repeats (STR) data from 24 Native American populations, a statistical evaluation of multiple models indicated that the data do not support models with only a single or two discrete migration waves and suggest a scenario with a more complicated continental migration [17]. However, a recently published paper suggested a three-wave model that the first wave migration contributed most of genetic components of Native Americans, except the Eskimo-Aleut speakers in the Arctic region and Na-Dene speakers from Canada [18]. Arguments about possible models for the initial colonization continue and more rigorous investigation will facilitate further clarification.

The history of population dispersion after the initial colonization of Native Americans is less discussed while the debates on the origins and the time of the first Americans receives the most attention of geneticists. Coastal routes of early American migrations have been proposed in both mtDNA and STR studies. Wang et al. suggested least-cost paths in a coastal migration scenario including both Pacific and Atlantic coastal routes [19]. Their Pacific coastal routes are well supported by the mtDNA evidence [8]. The times of the subsequent colonial events along these routes, however, are not known. An extended genetic study of the population history of Native Americans would provide comprehensive insight into the subsequent colonial events.

An understanding of the colonization and migration of Native Americans will depend on a good understanding of the genetic relationship of Native American populations. The genetic distance, a measurement of population divergence, is frequently applied to explore genetic affinity of human populations because the genetic distance has a linear relationship with the divergence time of pairwise populations in simple genetic scenarios [20]. A linear correlation between serial divergence times and classic pairwise genetic distances, however, may not hold in complicated demographic scenarios when multiple populations are involved and their effective population sizes vary drastically [21], [22]. A better approach that can reliably measure genetic affinity of populations in complicated genetic scenarios will greatly improve our understanding of the population genetics of Native Americans.

Microsatellites (or STR), one commonly used genetic marker, can be fitted well by a molecular clock, and can therefore provide a reliable estimate of the time of population divergence whereas even a large-scale single-nucleotide polymorphism (SNP) dataset cannot [23]. In the present study, we planned to take advantage of STR markers to explore the migratory and colonial history of Native American populations. Based on our theoretical work, we propose a novel genetic measure of the divergence of populations based on STR data. Computer simulations revealed that the novel measure maintained a good linear correlation with the divergence times of populations, even in complicated demographic scenarios, such as those related to the dispersion of Native Americans. By applying the novel measure to the STR data of 21 Native American populations [19], our investigation disclosed the genetic structure of these populations across vast geologic zones and suggests a rapid dispersion in Central and South America during the peopling of America.

## Results

### Evaluating the novel method using simulated STR data

A novel method was developed in this study to measure the genetic divergence of populations in complicated multi-population scenarios (see Methods for details). We addressed the performance of the novel method in two separate evaluations using simulated STR data.

**Figure 1:**
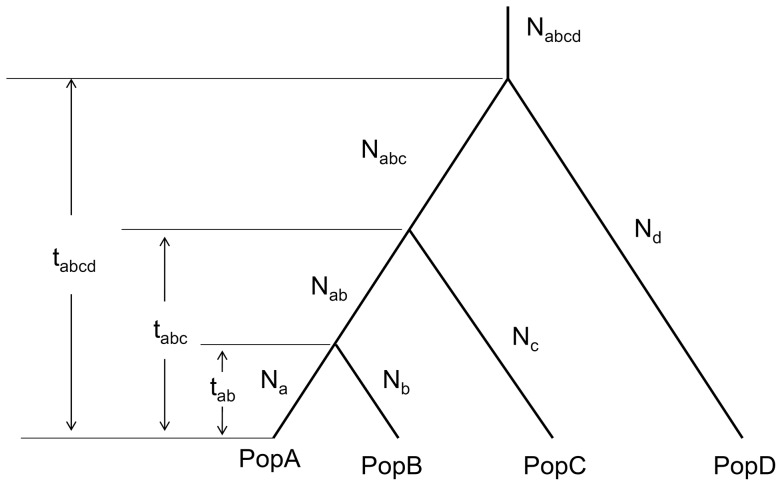
A four-population model with demographic parameters. Population PopD was the reference population with known divergence time when the model was used in our study.

**Figure 2:**
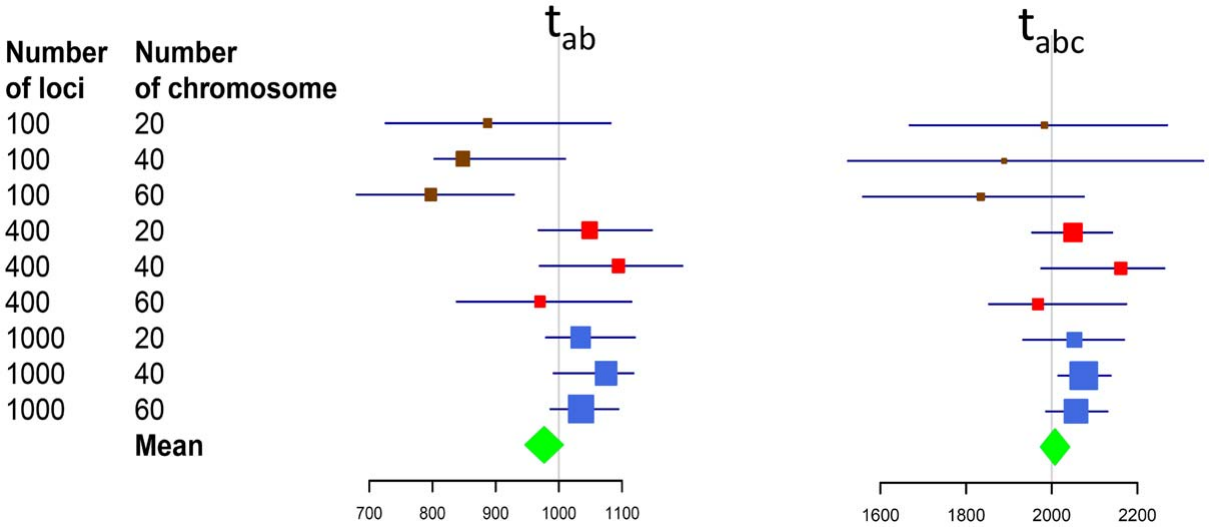
Medians with 95% CIs of 50 estimations for 9 different scenarios. Medians are presented in colored squares and their 95% CIs are shown as bars across each of the squares. The areas of the squares are in reverse proportion to their 95% CI. Diamonds indicate all the medians and the width of the diamond indicates the standard error of the medians. Vertical lines indicate pre-given values of the demographic parameters.

In the initial evaluation, performance of the novel method was examined for different sample sizes using 50 simulated STR datasets that were generated for a four-population demographic model (Figure 1, see Methods for details). Population divergence was estimated from each of the 50 simulated datasets. On each of the datasets, the novel method was tested multiple times with different loci numbers (100, 400, or 1000 loci) and different sample sizes (20, 40, or 60 chromosomes). The results showed that variance of the estimations decreased when more genetic loci were involved. The 95% confidence interval (CI) of the medians of estimations was significantly narrowed down with an increase in the loci number (Figure 2). The results suggested that our method provided estimates with a reasonable variance when the number of involved loci was larger than or equal to 400 (Figure 2). The results also indicated that sample size had little effect on the 95% CI. Therefore, our method performed nearly the same on genetic samples with 10 or 30 individuals. The mean of the medians of estimations was very close to pre-given population divergences, although the medians fluctuated (Figure 2). Hence, the simulations suggested that our method can be used to measure population divergence with little bias. More extended evaluations confirmed the above perspectives (data not shown).

**Figure 3:**
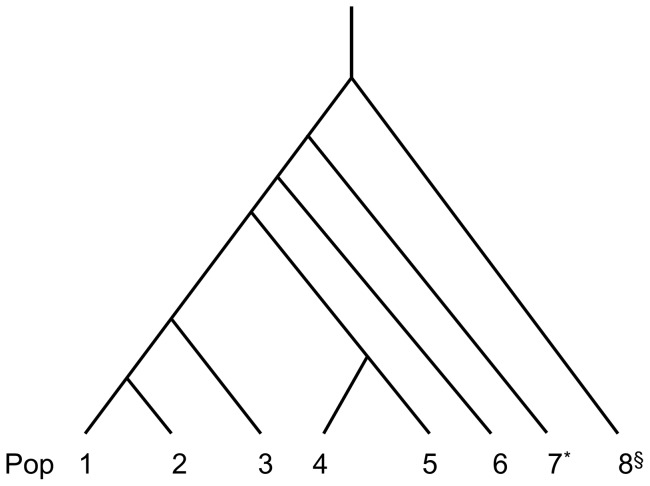
Scenario with 7 observed populations and 1 hidden population. The populations have current *Ne*, N_1_ = 1000, N_2_ = 2000, N_3_ = 4000, N_4_ = 6000, N_5_ = 2000, N_6_ = 10000, N_7_ = 10000, and N_8_ = 20000. Divergence times are given as T_12_ = 300, T_13_ = 500, T_14_ = 1000, T_45_ = 600, T_16_ = 1500, T_17_ = 2000, and T_18_ = 3000. The populations are also assumed to be in exponential growth with a constant rate of 0.001. *The population Pop7 was used as a reference for the evaluation. §The population Pop8 was assumed not to be observed in our study.

**Figure 4:**
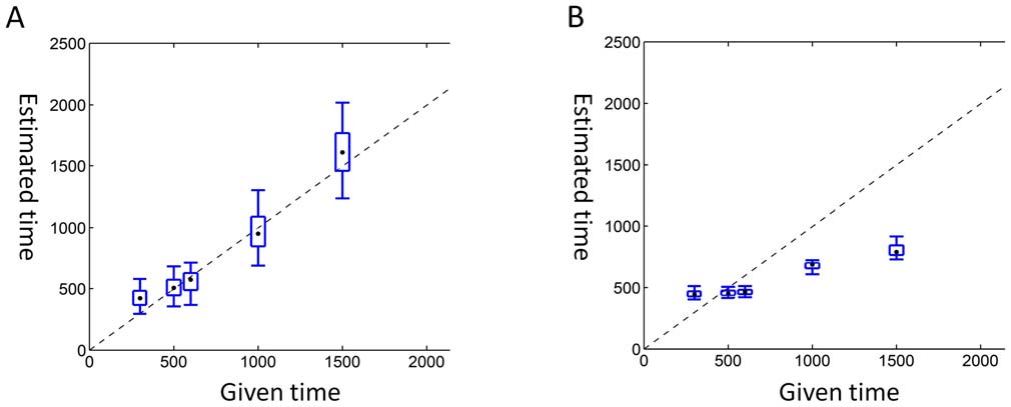
Box plots of the four-population approach and the existing method. A. Estimates from the four-population method show a linear relationship with the given values; B. Estimates from Goldstein’s method show a departure from the given values.

In further evaluation, the novel method was compared with Goldstein's method using simulated multi-population STR data [24]. The simulated data was generated from a complicate genetic model involving 8 populations (1 hidden population and 7 observed populations, Figure 3). All the populations were assumed to be expanding and with a different effective population size (*Ne,* see Methods for details). Five pairwise divergences among six observed populations (excluding the reference population) were estimated and the estimates were plotted against pre-given divergences using box plots (Figure 4). The estimates from our method were in good concordance with the pre-given divergences (Figure 4A). In comparison, the estimates from Goldstein’s method heavily departed from the pre-given divergences (Figure 4B). In extended evaluations for more genetic scenarios, our method consistently produced better estimates (with less bias and moderate variance) than that of Goldstein’s method, even when effective population sizes grew in non-exponential modes (data not shown). A serious bias would limit the application of Goldstein’s method in genetic studies involving multiple populations with size expansion, although the estimates from Goldstein’s method had smaller variances than those in our evaluations.

### Genetic divergences among Native Americans

**Figure 5:**
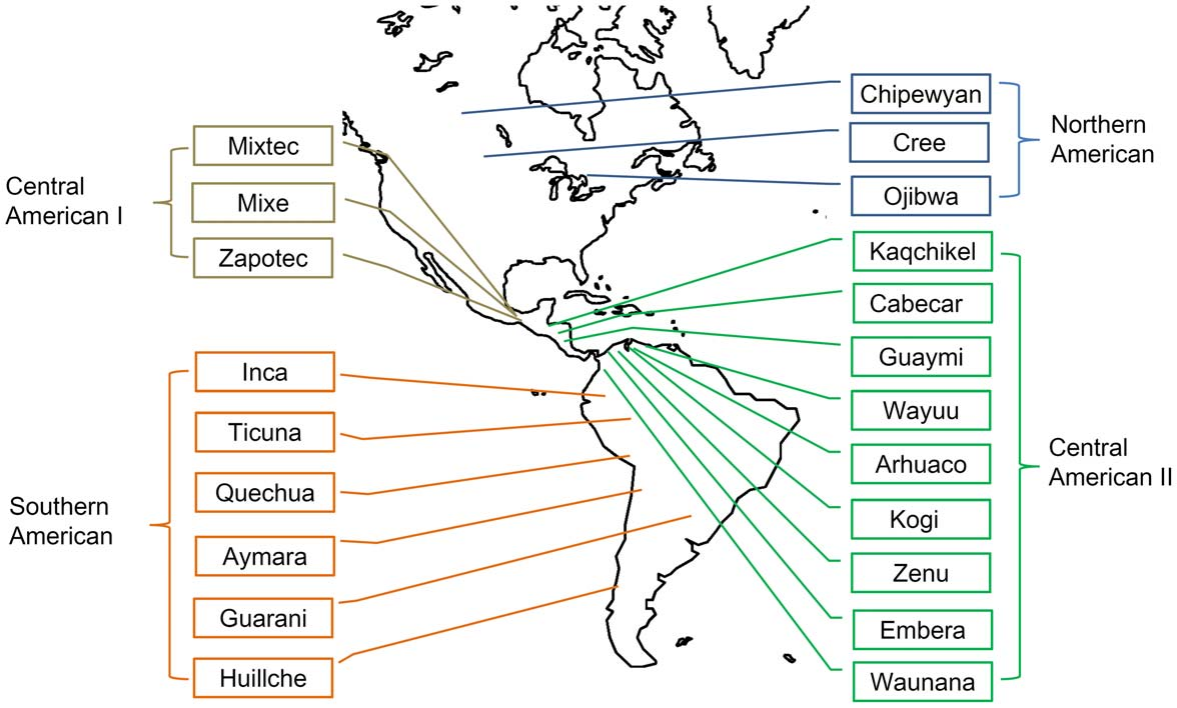
Geographic locations of 21 Native American populations. The populations are categorized into four different geographic clusters, i.e., Northern American, Central American I, Central American II, and Southern American.

**Figure 6:**
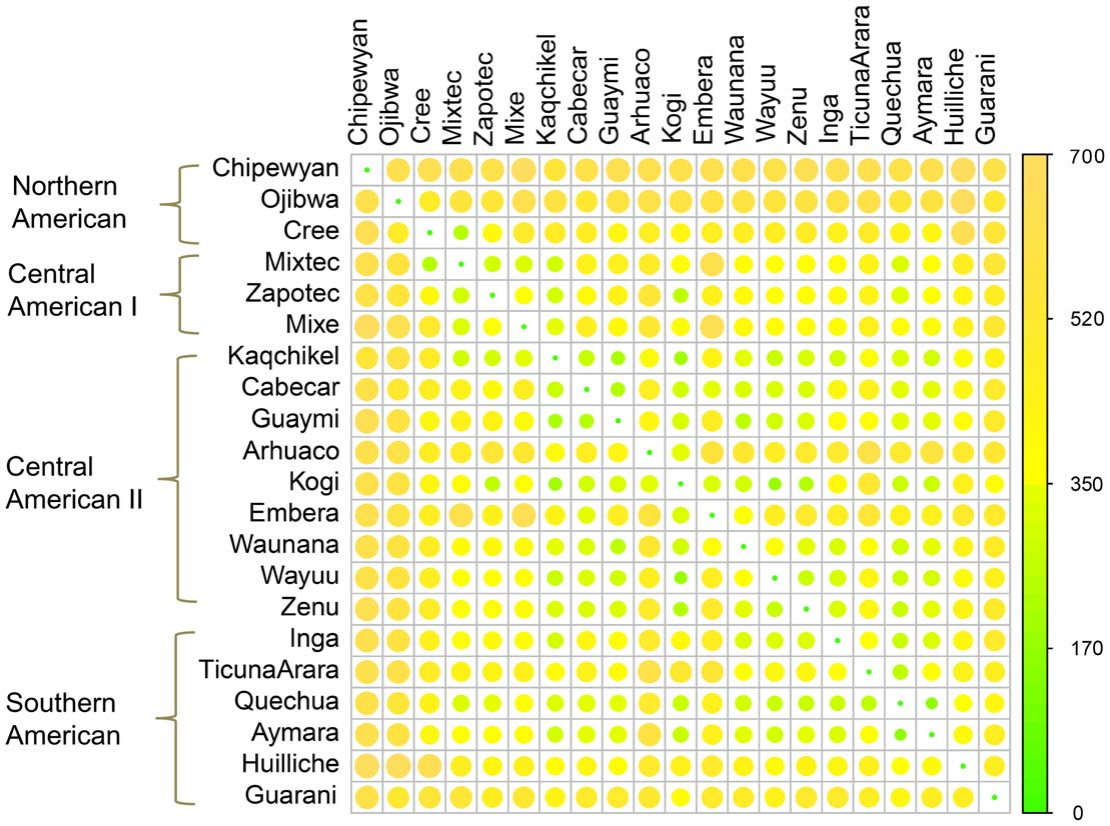
Pairwise divergences of 21 Native American populations. The size of the circles is proportionate to the value of the divergence. Color spectrum is given in the right bar.

Genetic divergences among Native Americans were investigated in this study using our novel method because of its competitive performance in multi-population scenarios. We applied the novel method on STR data of 21 Native American populations (Figure 5) and present their pairwise divergences in Table 1. The estimated divergences are also presented as a convenient visualization in Figure 6.

**Table 1:** Pairwise divergence of Native American populations.

	Chipewyan	Ojibwa	Cree	Mixtec	Zapotec	Mixe	Kaqchikel	Cabecar	Guaymi	Arhuaco	Kogi	Embera	Waunana	Wayuu	Zenu	Inga	TicunaArara	Quechua	Aymara	Huilliche	Guarani
**Chipewyan**	0	(618, 642)	(556, 654)	(630, 651)	(595, 615)	(647, 712)	(540, 554)	(583, 634)	(640, 680)	(597, 669)	(471, 646)	(605, 676)	(607, 679)	(595, 689)	(608, 691)	(605, 651)	(616, 665)	(602, 628)	(606, 631)	(598, 753)	(590, 630)
**Ojibwa**	628	0	(470, 487)	(551, 575)	(549, 567)	(582, 640)	(569, 593)	(502, 567)	(572, 611)	(555, 641)	(582, 617)	(559, 637)	(556, 640)	(552, 624)	(567, 640)	(562, 616)	(583, 721)	(539, 576)	(560, 599)	(548, 798)	(524, 563)
**Cree**	647	482	0	(231, 235)	(396, 409)	(503, 515)	(479, 487)	(458, 487)	(403, 427)	(420, 514)	(332, 500)	(417, 499)	(405, 498)	(406, 579)	(417, 506)	(414, 499)	(412, 519)	(428, 475)	(400, 465)	(458, 690)	(462, 567)
**Mixtec**	639	569	233	0	(237, 315)	(256, 351)	(221, 319)	(383, 495)	(369, 492)	(408, 552)	(315, 423)	(482, 699)	(318, 424)	(287, 389)	(314, 418)	(312, 429)	(391, 492)	(288, 352)	(300, 393)	(410, 500)	(474, 575)
**Zapotec**	603	559	399	292	0	(301, 391)	(238, 300)	(313, 438)	(365, 470)	(431, 599)	(191, 258)	(422, 509)	(349, 423)	(252, 356)	(285, 389)	(353, 420)	(372, 466)	(303, 354)	(298, 376)	(385, 428)	(418, 500)
**Mixe**	697	623	509	317	359	0	(270, 369)	(418, 511)	(363, 445)	(441, 590)	(330, 446)	(501, 712)	(349, 424)	(320, 449)	(341, 414)	(330, 394)	(426, 508)	(318, 411)	(324, 398)	(388, 490)	(459, 563)
**Kaqchikel**	548	584	483	285	281	323	0	(247, 321)	(185, 231)	(325, 430)	(164, 218)	(353, 482)	(236, 352)	(241, 300)	(227, 333)	(238, 331)	(349, 421)	(276, 336)	(241, 317)	(356, 394)	(389, 457)
**Cabecar**	607	528	474	456	398	461	279	0	(195, 244)	(422, 553)	(233, 299)	(277, 352)	(260, 339)	(255, 350)	(256, 362)	(350, 468)	(409, 540)	(280, 350)	(292, 400)	(374, 433)	(433, 550)
**Guaymi**	654	599	417	445	429	400	205	222	0	(369, 487)	(260, 328)	(401, 560)	(227, 313)	(251, 328)	(279, 377)	(313, 427)	(317, 400)	(269, 345)	(297, 400)	(303, 414)	(437, 559)
**Arhuaco**	623	601	476	499	556	522	394	476	420	0	(249, 402)	(514, 676)	(425, 603)	(394, 529)	(422, 583)	(434, 613)	(567, 657)	(405, 603)	(456, 631)	(454, 560)	(449, 576)
**Kogi**	607	593	412	378	241	368	192	282	303	338	0	(228, 360)	(226, 379)	(137, 211)	(172, 294)	(298, 487)	(429, 584)	(233, 366)	(230, 297)	(430, 483)	(279, 535)
**Embera**	628	594	478	637	460	652	434	326	480	577	308	0	(333, 525)	(441, 536)	(403, 539)	(411, 530)	(414, 584)	(370, 539)	(384, 511)	(456, 620)	(437, 565)
**Waunana**	630	590	475	371	389	387	323	310	252	523	290	379	0	(326, 394)	(315, 374)	(269, 373)	(306, 403)	(259, 330)	(279, 367)	(402, 468)	(362, 543)
**Wayuu**	619	579	479	349	341	363	280	308	287	468	170	478	350	0	(227, 306)	(291, 378)	(371, 438)	(233, 289)	(263, 335)	(358, 442)	(410, 491)
**Zenu**	644	593	491	375	375	380	301	305	309	518	226	504	339	270	0	(288, 381)	(363, 434)	(244, 298)	(278, 363)	(401, 470)	(439, 527)
**Inga**	631	593	455	367	381	371	288	403	371	514	387	480	309	313	318	0	(319, 420)	(221, 282)	(272, 358)	(401, 469)	(443, 543)
**TicunaArara**	631	613	487	436	428	445	370	455	369	604	535	553	345	419	409	348	0	(232, 290)	(300, 411)	(416, 499)	(373, 561)
**Quechua**	616	559	437	320	335	362	307	334	324	520	274	460	293	263	270	261	253	0	(122, 149)	(315, 368)	(356, 443)
**Aymara**	625	583	405	359	349	376	282	335	337	599	275	456	329	295	325	318	356	141	0	(358, 403)	(438, 486)
**Huilliche**	686	693	643	480	409	453	378	401	370	501	446	506	433	391	437	420	452	344	388	0	(430, 536)
**Guarani**	610	533	550	550	473	524	438	501	507	527	365	513	486	459	513	501	462	410	472	464	0

Estimates (median) are given in the lower left half of the table. The upper-right triangular part half of the table presents the first and third quartiles of the estimates.

Population divergences between a given population and all other populations are important for the historical timeline of the given population. In our investigation, three populations in North America had generally deep divergences from the remaining populations. Chipewyan, a North American population, had the greatest genetic divergence from the rest of the populations, ranging from 548 to 697 generations with a mean±s.d. of 628.65±30.82 generations (Table 1). The results support the notion that the Chipewyan population is one of the oldest branches of Native Americans. Ojibwa have a mean±s.d. divergence of 582.53±42.65 generations (Table 1). The divergences of the other North American populations: Cree, with mean±s.d. of 474.71±80.63 generations (excluding divergence between Cree and Mixtec), are younger than the Chipeywan and Ojibwa populations (Table 1). Interestingly, the divergence between Cree and Mixtec is only 233 generations (with first and third quartiles of 231 and 235 generations, respectively; Table 1). The small divergence suggests a close genetic relationship between the Cree and Mixtec. This close relationship is highly suspected to be a consequence of genetic admixture. Computer simulations confirmed that the genetic divergence of a mixed population could be underestimated due to genetic admixture (Text S1).

Among the Central and South American populations, pairwise divergences varied with a mean±s.d. of 377.81±101.47 generations (Table 1). The divergences were much smaller than that of the North America populations. The Arhuaco and Guarani populations showed prominent divergences from the other Central and South American populations. The Arhuaco peoples living in the mountainous area of northern Colombia showed pairwise divergences with mean±s.d. of 498.5±72.71 generations. The Guarani (residing in Southern Brazil) had divergences with a mean±s.d. of 472.71±44.95 generations from the other Central and South Americans (Table 1).

The maximum pairwise divergence was 697 generations between the Mixe and Chipewyan populations (Table 1). The Chipewyan language is a member of the Na-Dene language family and Mixe Americans are Amerind speakers [25]. The Na-Dene language is linked with the Yeniseian languages of Siberia, whereas most Native American languages are members of the Amerind family [26]. The minimum divergence was only 141 generations, between the Quechua and Amyra populations. The two populations lived in the Pacific Rim of South America and their languages belong to branches of the Andean stock of the Amerind family.

### Population groups & divergences of groups

**Figure 7:**
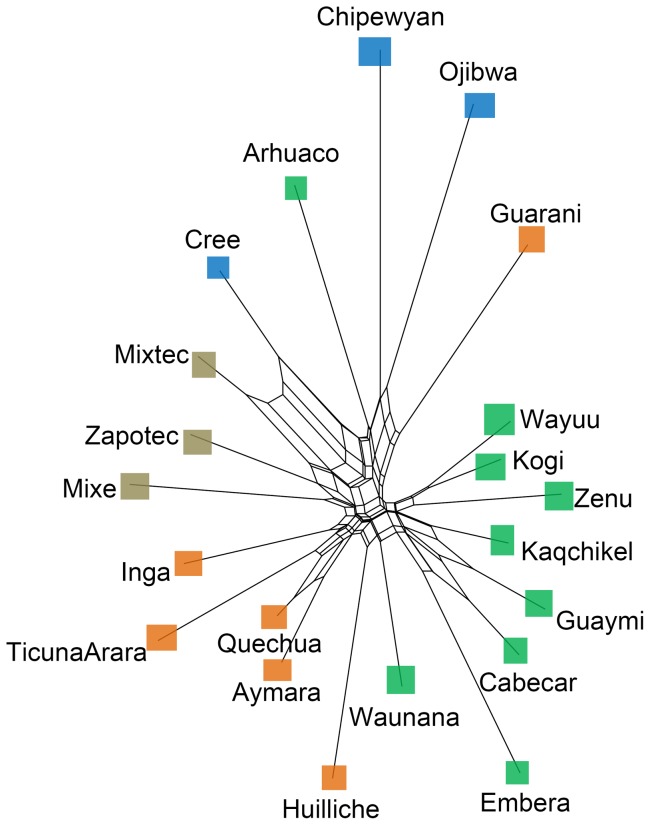
Neighbor-Net of 21 Native American populations. The color of the squares indicates the geographic relationship of the populations (see Figure 5 for more details).

The genetic relationship of the populations was further explored using Neighbor-Net because the Neighbor-Net method is more powerful than the Neighbor-joining tree method (Figure 7) [27]. By combining information from both the structure of the reconstructed network and geographic locations of the populations, Native Americans could be categorized into four groups, except for the aforementioned Arhuaco and Guarani populations (Figure 5). The first group included three Northern American populations, Chipeywan, Ojibwa, and Cree. Zapotec, Mixtec, and Mixe comprised the second group (Central American I). The remaining populations in Central America and nearby formed the third genetic group (Central American II, including Kaqchikel, Cabecar, Guaymi, Kogi, Embera, Waunana, Wayuu, and Zenu). The populations living in Pacific Rim of South America (Inga, Ticuna Arara, Quechua, Aymara, and Huilliche), mainly in the area of the Andes Mountains, comprised the fourth group (Southern Americans). Historical procedures for forming the four population groups may be considered as steps of the early colonization of human beings in America because the route of early immigration in the Pacific Rim crossed sequentially through the living areas of the four population groups [8].

**Figure 8:**
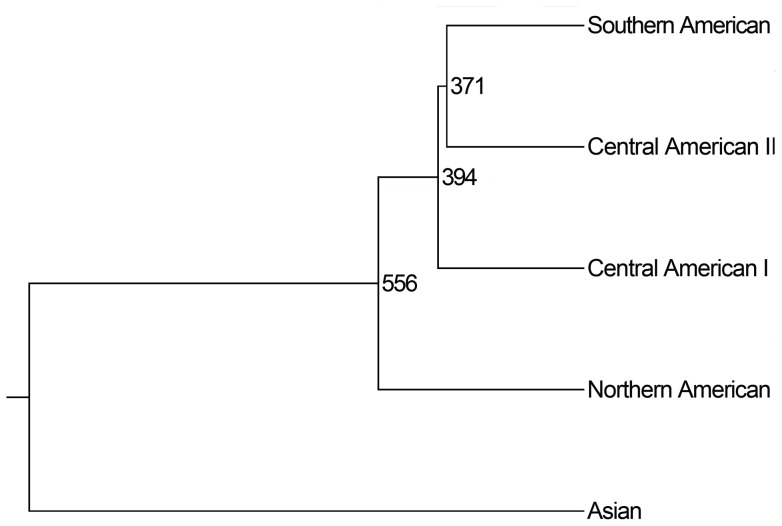
UPGMA tree of four population groups of Native Americans.

To evaluate the serial colonization events in America, we calculated mean population divergences between the four population groups (Table 2). An unweighted pair group method with arithmetic mean (UPGMA) tree based on the group divergences presents the possible colonial process of human beings in the New World (Figure 8). After the initial colonization in North America, the first wave of population stratification might have occurred in 556 generations BP. Some of the early immigrants moved toward Central America. After a standstill of about 162 generations, the second wave of population stratification occurred (394 generations BP). Native Americans migrated to the far southern part of North America. Some of them passed over the land bridge to South America. In only 23 generations, the early inhabitants of South America dispersed to distant areas of South America (371 generations BP), mainly along the Andes Mountains.

**Table 2:** Divergences among four population groups. The results are presented in generations.

		[1]	[2]	[3]	[4]
[1]	Northern American				
[2]	Central American I	536.78			
[3]	Central American II	554.42	397.67		
[4]	Southern American	577.13	391.40	371.13	

## Discussion

The findings of the present study showed that the proposed method provided good estimates of population divergence (Figures 2&4). Therefore, the estimates of Native American populations in this study should provide more details about the peopling of the Americas, especially the peopling of South America.

The Clovis culture appeared in many sites of North America about 13,000 years BP, while the oldest remains of human activity are at least 18,000 to 22,000 years old [28], suggesting that humans entered North America long before the Clovis Culture. Therefore, it is reasonable conjecture that any population arriving in America in the second-wave of immigration or later should show substantial differentiation from the first-wave immigrants. The divergence between the North American populations and South American populations in this study was only 556 generations BP (Figure 8). The divergence was about 11,120 to 13,900 years old when the duration of a generation was assumed to be 20 to 25 years. The split between the two population groups, therefore, is not likely deep enough to support the hypothesis that their ancestors arrived in America in significantly different migration waves. It should be mentioned, however, that our study did not eliminate the possibility of a two- or multi-wave model with an "equal admixture" or "complete replacement" before the population divergence. Furthermore, our study did not eliminate the possibility of a two- or multi-wave model that only one of the migration waves contributed nearly all of genetic composition in populations. A recently published paper suggested that the one wave migration contributed most of genetic components of Native Americans, except the Eskimo-Aleut speakers in the Arctic region and Na-Dene speakers from Canada [18].

A population admixture could decrease divergence of the involved populations. In other words, a split between the involved populations could be much older than the estimate if genetic communication occurred between the populations. Thus, the population divergence estimate may fail to disclose two- or multi-wave immigration in early colonization in the admixture scenario. In our study of population group divergence, however, the impact of admixture is expected to be limited because the two aforementioned population groups lived far from each other, thousands of miles away, and geographic space could act as an efficient barrier of gene flow [29], [30]. Potential genetic communication is more likely to happen within population groups, but to be lower between populations from distinct population groups. The theory of 'isolation by distance' in Native Americans was partially evaluated in a study that examined genetic heterozygosity and least-cost paths in a coastal migration scenario [19]. Furthermore, extended computer simulations showed that minor gene influx would have only a limited effect on our divergence measure (Text S1). It was probably due to the negligible impact of minor gene influx on the expected coalescent time of the two lineages.

The European contribution was generally concerned with genetic studies of Native Americans when the genetic contribution was not likely to be significant in Native Americans, even for populations living in North America [19], [31]. The results in the present report are still reliable in a scenario with a limited European gene influx because of the aforementioned robustness of the novel method to minor genetic admixture (Text S1). A recent study suggested that the European contribution might be as much as ∼40% in the North American population [32]. That study, however, probably highly overestimates the contribution of Europeans to Native Americans because the result conflicted with a previous report based on similar analysis and the same STR data. If a significant European contribution was true, however, our results and many previously published results should be reconsidered with a genetic scenario with a non-negligible European component.

Our analysis suggests the Native Americans had a long-time standstill in North America before some of them stratified and moved into South America. The exact duration of the standstill is not known, but its lower bound is estimated to be 162 generations (about 3240–4050 years, Figure 8). This estimate is consistent with a previous report suggesting a long-time standstill in the Bering area [33].

Estimates of divergences of the population groups suggested that Native Americans might have reached South America in 394 generation BP (about 7890–9863 years ago, Figure 8). The estimates are well supported by archeological evidence. The earliest known skeletal evidence in South America is dated to almost 8500 to 10,000 years BP [34]–[37]. Our results also indicated that Native Americans took only 23 generations (468–585 years) to get across the land bridge (Figure 8). The widespread distribution of the fishtail point (a type of stone artifact) and its variants among archeological sites of Central and South America support the period of rapid movement of the population or diffusion [37].

A total of 21 Native American populations were involved in this study. Limited genetic data is a main hindrance to the current investigations of the genetic history of Native Americans. All published perspectives should be further evaluated with multi-source genetic data from more populations in the future.

## Materials and Methods

### Brief summary of previous study

Slatkin showed the relationship between size variance of STR and coalescent time of pairwise lineages [38]. Suppose two copies of the same STR marker were sampled randomly from a population or two different populations and the sizes of the two alleles were S_1_ and S_2_. The size difference of the two copies changes with an accumulation of mutations after the divergence of the two lineages. If the change in allele size due to mutation *i* is *x_i_*, the size difference of the two alleles could be presented as
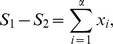
where α is the total number of mutations that accumulated after divergence of the two lineages. In the stepwise mutation model, each *x_i_* is independent of the other with a mean of 0 and variance σ^2^, and the variance of the size difference could be presented as



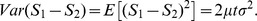
When the STR data of *m* independent loci is available, the expected coalescent time of the two lineages is estimated as



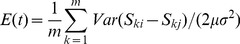
(1)


### Our model and solution for divergence time estimation

#### I. Relationship of demographic parameters in a two-population model

**Figure 9:**
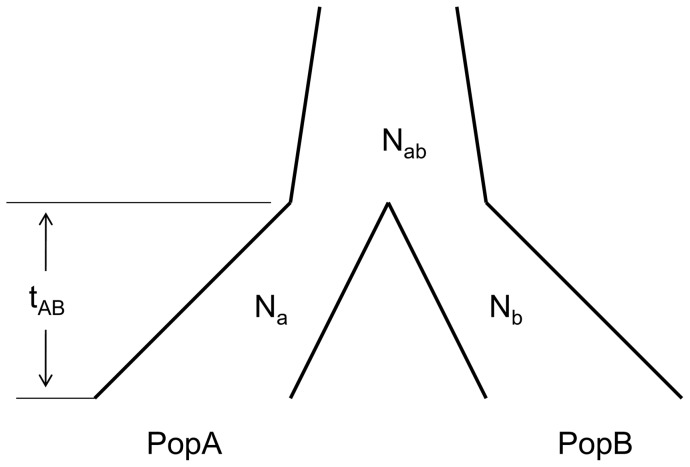
A two-population model with population growth.



To present the relationship of coalescent time of lineages and the populations’ divergence time in a Wright-Fisher model with two populations (Figure 9), we assume that populations grow exponentially (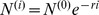, where r is the increasing factor and *i* is the time from present) and effective population size (counting in number of effective chromosome, *Ne* in text and *N* in equations) of the ancestral population (Pop_ab_) is the sum of the newborn populations’ *Ne* at divergence time t (namely, 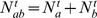 at divergence time *t*). Therefore, we have the equations below:

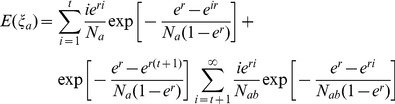
(2)




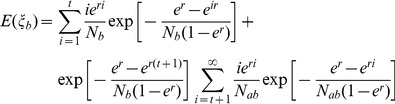
(3)




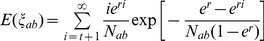
(4)where *E(ξ_a_)*, *E(ξ_b_)*, and *E(ξ_ab_)* are expectations of coalescent times of pairwise lineages within population A, within population B, and between populations A and B, respectively. Equations (2)–(4) can be used to estimate demographic parameters (including population divergence time) when the expected coalescent times of two lineages within or between populations are available from Equation (1).

**Figure 10:**
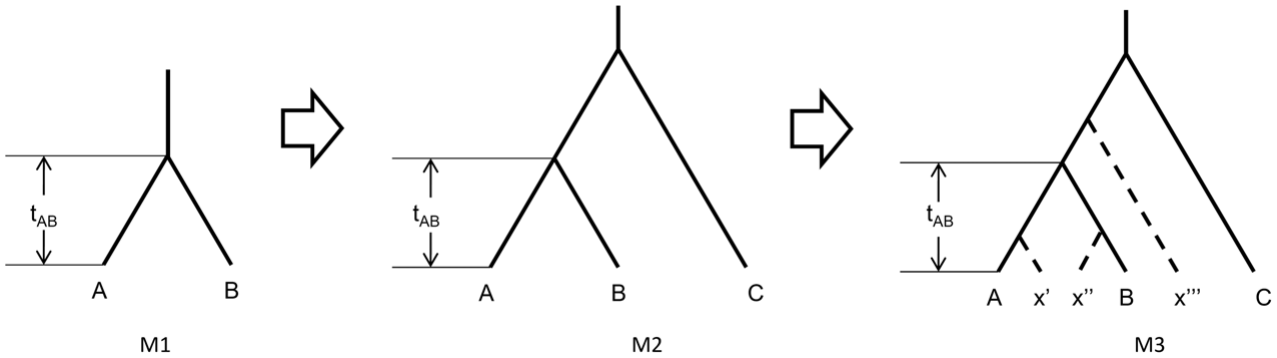
Moving from the two-population model to a four-population model.

Using Equations (2)–(4), three of the four demographic parameters (*N_a_*, *N_b_*, and *t_AB_*) in a two-population model can be calculated (Figure 10, Model M1) if the population growth rate (*r*) and expectations of the coalescent times (*E(ξ_a_)*, *E(ξ_b_)*, and *E(ξ_ab_)*) are known. In most studies however, the population growth rate is not known. To solve this problem, we introduced a reference population *c* with a known divergence time (Figure 10, Model M2). Following the aforementioned relationships among demographic parameters in the Wright-Fisher model, we could build a system of equations (including 5 equations) for model M2 to calculate *t_AB_* and other unknown demographic parameters (*N_a_, N_b_, N_c_*, and r) simultaneously (results not shown).

#### II. Extension to a four-population model

It is trivial to develop a model to include all populations when there are more than 20 Native American populations involved in our study. Therefore, we aimed to propose an explicit method that could be easily applied in scenarios with many populations. Introduction of any extra populations to model M2 (beyond Populations A, B, and C), however, will affect the estimation of *t_AB_* because *Ne* of the ancestral population at the time of divergence is the sum of all the newborn populations’ *Ne* in our models. We moved from model M2 to model M3, therefore, to evaluate the impact of an extra population X on the estimation of *t_AB_* (Figure 10, Model M3). It was assumed that population X appeared in Positions x’, x’’, or x’’’ (Figure 10, Model M3). In a multi-population scenario (more than 4 populations), we obtained different estimates (values) of divergence *t_AB_* when different populations were introduced as population X. The median of the serial estimates (values) was used as a robust estimate of the divergence *t_AB_* and distribution of the serial values supplied information about the reliability of the indicators. In other words, a four-population model (including Populations A, B, C, and X) could be applied to estimate population divergence when multiple populations were involved.

#### III. Calculating divergence in a four-population or multi-population scenario

Model M3 could be transformed into a universal four-population model when we reassigned symbols (A, B, C, and D) to the four populations (see Figure 3) because the three possible four-population trees of model M3 had the same topological structure regardless of where the actual position of the population X is.

Following the same principles as that of Equations (2)–(4), we constructed a system of equations for the aforementioned four-population model (Figure 1). The system includes seven equations corresponding to seven expectations of coalescent times of pairwise lineages within or between the four populations (Text S2). Roots of the equations are estimations of unknown demographic parameters in the four-population model (Figure 1). The system of equations can be solved by the damped Newton-Raphson method with the constraint that all roots of the equations are larger than or equal to zero [39].

Demographic parameters for 21 Native American populations were estimated in a two-stage procedure in this report. First, we estimated the population demographic parameters for all four-population combinations, including the Asian population reference and three other Native American populations. After that, the median of all the estimated divergences between each pair of Native American populations was used as a robust estimation of divergences between the paired populations.

#### IV. Performance evaluation

We evaluated the aforementioned method in two separate assessments using simulated STR data. The simulated STR data were produced from simulated sequences from MS using a strict stepwise mutation model [40], [41]. The mutation rate of the STR was assumed to be 6.4×10^−4^ [42]. STR loci were independent in our simulations.

In the first assessment, we examined the performance of the system of equations and the equation solver in the genetic model with only four populations (Figure 1). In the simulation, effective population sizes of the four populations were given as 4000, 2000, 6000, and 8000 for populations PopA, PopB, PopC, and PopD, respectively, when divergence times were assumed to be 1000, 2000, and 3200 generations for *t_AB_*, *t_ABC_*, and *t_ABCD_*, respectively. The population growth rate was set to 0.001 in the simulations. The evaluation was carried out with a different sample size (20, 40, or 60 chromosomes) and different locus number (100, 400, or 1000 loci) on a total of 50 independent simulated data sets. Estimation bias was cautiously addressed using medians of 50 estimates and overall means of the medians. The 95% CI of the medians was used to assess the reliability of the estimates for each different sample size and locus number (Figure 2).

In the second assessment, our method was evaluated in a more complicated scenario with one hidden population and seven observed populations. Pre-given demographic parameters for STR simulation were presented with the population model in Figure 3. Using the aforementioned solution for a multi-population condition, the divergence times of six observed populations were estimated when the oldest observed population was used as a reference. Simultaneously, a frequently-used method from Goldstein et al. was also applied to the same simulation data to estimate the divergence times of the pairwise populations [24]. Pre-given divergence times of the six populations and estimated times from both the two different estimating methods in box plots are shown in Figure 4 for direct comparison. More demographic parameters and models were evaluated in the extended evaluations.

### Genetic data

STR data from Wang et al. was used in this study [19]. The full data set included genotypes of 678 autosome STR markers from 436 individuals. The samples were collected from 24 Native American populations and one Asian population from Central Siberia.

We transformed data of all alleles into numbers of repeats. Only tetra-repeat loci were further investigated. Finally, 451 tetra-repeat loci were involved in our analysis with missing data less than or equal to 10% per locus. Genetic relatives were detected in the primary data set (named N436). Wang et al. excluded the relatives to reform data sets N379 and N354 [19]. We analyzed all three data sets for a full view of population divergences. Ache, Kaingang, and Ticuna (Tarapaca) were excluded from the final analysis because these populations had larger within-population divergences than between-population divergences, which might be due to population admixture, sampling error, or random factors. We presented population names and geographic locations of the remaining 21 Native American populations in Figure 5.

### Exploring divergences of Native American populations

Expected coalescent times of pairwise lineages within or between populations were calculated using Equation (1) in this study. We used a mean mutation rate μ = 6.4×10^−4^ per generation for the involved tetra-repeat loci [42]. The Tundra Nentsi population from Central Siberians was considered as a population reference because of its geographic location.

Using Zhivotovsy’s methods, the empirical value of divergence time between Native Americans and Siberians (Tundra Nentsi population) was estimated to be 1536 generations with the upper bound 3721 generations [22]. In the estimation, mean STR size variance of three gather-hunter populations (Ache, Karitiana, and Surui) was used as that of common ancestral population of Native Americans. The empirical estimation was concordant with archaeologic and mtDNA studies, suggesting a general range of 20 to 30 kyr BP for the first colonization of America [8], [28], [43]. Therefore, we used 1500 generations as the divergence time between Asian references and Native American populations in further analysis.

Pairwise divergences of populations were estimated using the aforementioned multi-population method. For each population pair, we obtained 19 divergence values (estimation) in all, corresponding to calculations with different interference populations (Population X in model M3). A median of 19 results was used as the final estimate for divergence. The first and third quartiles of the 19 results supplied information for reliability of the final result. Neighbor-Net was constructed on the basis of the estimated divergences to present the genetic relation of the Native American populations. The Neighbor-Net caught more details than the tree-based presentation [27].

To further eliminate the uncertainty of the estimations, we divided the 21 populations into 4 population groups according to their genetic affinity and geographic locations. Divergence of the population groups was defined as the mean divergences of between-group population pairs. We constructed a UPGMA tree for the divergences of groups to present a historical pattern of sequential colonial events in the geographic regions.

## Supporting Information

Text S1 Effects of genetic admixture on the novel method.
(PDF)

Text S2 A system of equations based on the four-population model.
(PDF)

## References

[1] Stoneking M, Delfin F (2010) The human genetic history of East Asia: weaving a complex tapestry. Curr Biol 20: R188–193 doi:10.1016/j.cub.2009.11.052. 20178766 10.1016/j.cub.2009.11.052

[2] Soares P, Achilli A, Semino O, Davies W, Macaulay V, et al (2010) The archaeogenetics of Europe. Curr Biol 20: R174–183 doi:10.1016/j.cub.2009.11.054. 20178764 10.1016/j.cub.2009.11.054

[3] Majumder PP (2010) The human genetic history of South Asia. Curr Biol 20: R184–187 doi:10.1016/j.cub.2009.11.053. 20178765 10.1016/j.cub.2009.11.053

[4] Campbell MC, Tishkoff SA (2010) The evolution of human genetic and phenotypic variation in Africa. Curr Biol 20: R166–173 doi:10.1016/j.cub.2009.11.050. 20178763 10.1016/j.cub.2009.11.050 PMC2945812

[5] O’Rourke DH, Raff JA (2010) The human genetic history of the Americas: the final frontier. Curr Biol 20: R202–207 doi:10.1016/j.cub.2009.11.051. 20178768 10.1016/j.cub.2009.11.051

[6] Perego UA, Angerhofer N, Pala M, Olivieri A, Lancioni H, et al (2010) The initial peopling of the Americas: a growing number of founding mitochondrial genomes from Beringia. Genome Res 20: 1174–1179 doi:10.1101/gr.109231.110. 20587512 10.1101/gr.109231.110 PMC2928495

[7] Dillehay TD (2009) Probing deeper into first American studies. Proc Natl Acad Sci USA 106: 971–978 doi:10.1073/pnas.0808424106. 19164556 10.1073/pnas.0808424106 PMC2633551

[8] Fagundes NJR, Kanitz R, Eckert R, Valls ACS, Bogo MR, et al (2008) Mitochondrial population genomics supports a single pre-Clovis origin with a coastal route for the peopling of the Americas. Am J Hum Genet 82: 583–592 doi:10.1016/j.ajhg.2007.11.013. 18313026 10.1016/j.ajhg.2007.11.013 PMC2427228

[9] Haynes G (2002) The early settlement of North America: the Clovis era. Cambridge: Cambridge Univ Press. 345 p.

[10] Bonatto SL, Salzano FM (1997) A single and early migration for the peopling of the Americas supported by mitochondrial DNA sequence data. Proc Natl Acad Sci USA 94: 1866–1871. 9050871 10.1073/pnas.94.5.1866 PMC20009

[11] Merriwether DA, Rothhammer F, Ferrell RE (1995) Distribution of the four founding lineage haplotypes in Native Americans suggests a single wave of migration for the New World. Am J Phys Anthropol 98: 411–430 doi:10.1002/ajpa.1330980404. 8599378 10.1002/ajpa.1330980404

[12] Forster P, Harding R, Torroni A, Bandelt HJ (1996) Origin and evolution of Native American mtDNA variation: a reappraisal. Am J Hum Genet 59: 935–945. 8808611 PMC1914796

[13] Zegura SL, Karafet TM, Zhivotovsky LA, Hammer MF (2004) High-resolution SNPs and microsatellite haplotypes point to a single, recent entry of Native American Y chromosomes into the Americas. Mol Biol Evol 21: 164–175 doi:10.1093/molbev/msh009. 14595095 10.1093/molbev/msh009

[14] Greenberg JH, Turner CG, Zegura SL (1986) The settlement of the Americas: A comparison of the linguistic, dental, and genetic evidence. Curr Anthropol 27: 477–497.

[15] Kitchen A, Miyamoto MM, Mulligan CJ (2008) A three-stage colonization model for the peopling of the Americas. PLoS ONE 3: e1596 doi:10.1371/journal.pone.0001596. 18270583 10.1371/journal.pone.0001596 PMC2223069

[16] Mulligan CJ, Kitchen A, Miyamoto MM (2008) Updated three-stage model for the peopling of the Americas. PLoS ONE 3: e3199 doi:10.1371/journal.pone.0003199. 18797500 10.1371/journal.pone.0003199 PMC2527656

[17] Ray N, Wegmann D, Fagundes NJR, Wang S, Ruiz-Linares A, et al (2010) A statistical evaluation of models for the initial settlement of the american continent emphasizes the importance of gene flow with Asia. Mol Biol Evol 27: 337–345 doi:10.1093/molbev/msp238. 19805438 10.1093/molbev/msp238

[18] Reich D, Patterson N, Campbell D, Tandon A, Mazieres S, et al.. (2012) Reconstructing Native American population history. Nature. Epub 11 July 2012. doi:10.1038/nature11258. 10.1038/nature11258 PMC3615710 22801491

[19] Wang S, Lewis CM, Jakobsson M, Ramachandran S, Ray N, et al (2007) Genetic variation and population structure in native Americans. PLoS Genet 3: e185 doi:10.1371/journal.pgen.0030185. 18039031 10.1371/journal.pgen.0030185 PMC2082466

[20] Weir BS, Cockerham CC (1984) Estimating F-statistics for the analysis of population structure. Evolution 38: 1358–1370. 28563791 10.1111/j.1558-5646.1984.tb05657.x

[21] Beaumont MA (1999) Detecting population expansion and decline using microsatellites. Genetics 153: 2013–2029. 10581303 10.1093/genetics/153.4.2013 PMC1460853

[22] Zhivotovsky LA (2001) Estimating divergence time with the use of microsatellite genetic distances: impacts of population growth and gene flow. Mol Biol Evol 18: 700–709. 11319254 10.1093/oxfordjournals.molbev.a003852

[23] Sun JX, Mullikin JC, Patterson N, Reich DE (2009) Microsatellites are molecular clocks that support accurate inferences about history. Mol Biol Evol 26: 1017–1027 doi:10.1093/molbev/msp025. 19221007 10.1093/molbev/msp025 PMC2734136

[24] Goldstein DB, Ruiz Linares A, Cavalli-Sforza LL, Feldman MW (1995) Genetic absolute dating based on microsatellites and the origin of modern humans. Proc Natl Acad Sci USA 92: 6723–6727. 7624310 10.1073/pnas.92.15.6723 PMC41401

[25] Diamond J (2011) Linguistics: Deep relationships between languages. Nature 476: 291–292. 21850102 10.1038/476291a

[26] Lewis MP (2009) Ethnologue: Languages of the world. 16th ed. Dallas: SIL International. 1248p.

[27] Bryant D, Moulton V (2004) Neighbor-net: an agglomerative method for the construction of phylogenetic networks. Mol Biol Evol 21: 255–265 doi:10.1093/molbev/msh018. 14660700 10.1093/molbev/msh018

[28] Goebel T, Waters MR, O’Rourke DH (2008) The late Pleistocene dispersal of modern humans in the Americas. Science 319: 1497–1502 doi:10.1126/science.1153569. 18339930 10.1126/science.1153569

[29] Barton NH (2008) The effect of a barrier to gene flow on patterns of geographic variation. Genet Res 90: 139–149 doi:10.1017/S0016672307009081. 10.1017/S0016672307009081 18289408

[30] Gayden T, Cadenas AM, Regueiro M, Singh NB, Zhivotovsky LA, et al (2007) The Himalayas as a directional barrier to gene flow. Am J Hum Genet 80: 884–894 doi:10.1086/516757. 17436243 10.1086/516757 PMC1852741

[31] Williams RC, Knowler WC, Pettitt DJ, Long JC, Rokala DA, et al (1992) The magnitude and origin of European-American admixture in the Gila River Indian Community of Arizona: a union of genetics and demography. Am J Hum Genet 51: 101–110. 1609790 PMC1682879

[32] Hunley K, Healy M (2011) The impact of founder effects, gene flow, and European admixture on native American genetic diversity. Am J Phys Anthropol 146: 530–538 doi:10.1002/ajpa.21506. 21913174 10.1002/ajpa.21506

[33] Tamm E, Kivisild T, Reidla M, Metspalu M, Smith DG, et al (2007) Beringian standstill and spread of Native American founders. PLoS ONE 2: e829 doi:10.1371/journal.pone.0000829. 17786201 10.1371/journal.pone.0000829 PMC1952074

[34] Neves W, Meyer D, Pucciarelli H (1993) The contribution of the morphology of early South and North American skeletal remains to the understanding of the peopling of the Americas. Am J Phys Anthropol 16: 150–151.

[35] Stothert KE (1985) The preceramic Las Vegas culture of coastal Ecuador. Am Antiq 50: 613–637.

[36] Dillehay TD, Calderón GA, Politis G (1992) Earliest hunters and gatherers of South America. J World Prehis 6: 145–204.

[37] Dillehay TD (1999) The late Pleistocene cultures of South America. Evol Anthropol 7: 206–216.

[38] Slatkin M (1995) A measure of population subdivision based on microsatellite allele frequencies. Genetics 139: 457–462. 7705646 10.1093/genetics/139.1.457 PMC1206343

[39] Galantai A (2000) The theory of Newton’s method. J Comput Appl Math 124: 25–44.

[40] Valdes AM, Slatkin M, Freimer NB (1993) Allele frequencies at microsatellite loci: the stepwise mutation model revisited. Genetics 133: 737–749. 8454213 10.1093/genetics/133.3.737 PMC1205356

[41] Hudson RR (2002) Generating samples under a Wright-Fisher neutral model of genetic variation. Bioinformatics 18: 337–338. 11847089 10.1093/bioinformatics/18.2.337

[42] Zhivotovsky LA, Rosenberg NA, Feldman MW (2003) Features of evolution and expansion of modern humans, inferred from genomewide microsatellite markers. Am J Hum Genet 72: 1171–1186 doi:10.1086/375120. 12690579 10.1086/375120 PMC1180270

[43] Kumar S, Bellis C, Zlojutro M, Melton PE, Blangero J, et al (2011) Large scale mitochondrial sequencing in Mexican Americans suggests a reappraisal of Native American origins. BMC Evol Biol 11: 293 doi:10.1186/1471–2148-11-293. 21978175 10.1186/1471-2148-11-293 PMC3217880

