# Supplementary material for: Genetic Divergence Disclosing a Rapid Prehistorical Dispersion of Native Americans in Central and South America
Source: PLoS One. 2012 Sep 6;7(9):e44788. doi: 10.1371/journal.pone.0044788 (PMC3435283; doi:10.1371/journal.pone.0044788)

Text S1 Effects of genetic admixture on the novel method

Computer simulations were conducted to evaluate impacts of genetic admixture on our method. A genetic model presented in the manuscript (Figure 3) was improved to accommodate genetic admixture between populations. 60 haploid genomes with 500 STR loci were sampled from each of the populations and used for two separate evaluations. In the first evaluation, STR data were simulated in two different scenarios that 6 or 20 per 60 haploid genomes from the population Pop1 (receptor) were actual legacy of population Pop4 (donor); in the second evaluation, 6 or 20 per 60 haploid genomes from each of the population Pop1 and Pop4 (receptors) were assumed to be genome copies from population Pop8 (donor) in two different scenarios, respectively. The contributions of gene influx are about 10% and 33.3% for genetic admixture with 6 or 20 per 60 haploid genomes, respectively.

In the first evaluation, estimates from our method were still in a good linear relationship with the expected divergence except that divergence between population Pop1 and Pop4 was slightly underestimated (Figure S1). The underestimation was more significant when gene influx increased from 6 to 20 per 60 haploid genomes (Figure S1 D). In the second evaluation (Figure S1), linear relationship between estimates and expectations is generally acceptable in scenario with only minor genetic influx (6 per 60 haploid genomes, Figure S2 B). However, performance of the novel method became much worse when genetic influx increased to 20 per 60 haploid genomes. Variance of the estimates greatly increased even if the linear relationship between expectations and estimates are still much better than that of Goldstein's estimations (Figure S2 D). Poor performance in the scenario with significant genetic admixture should be largely due to that the simulated demographic scenario was greatly different from the working model of the novel method (see Methods for details of the working model).

To summary, our method is robust to minor genetic admixture and gene influx from genetic donor to receptor leads to an underestimation to their divergence.

Figure S1 Box plots for effects of gene influx from the population Pop4 to Pop1. Expected population divergence was given on Y-axis and estimate of population divergence was marked on X-axis. The results for scenario with a gene influx of 6 per 60 haploid genomes were showed on Figure S1 A&B; the results for scenario with a gene influx of 20 per 60 haploid genomes were presented in Figure S1 C&D. The Figure S1 A&C presented results of Goldstein's method; the Figure S1 B&D presented results from the novel method.

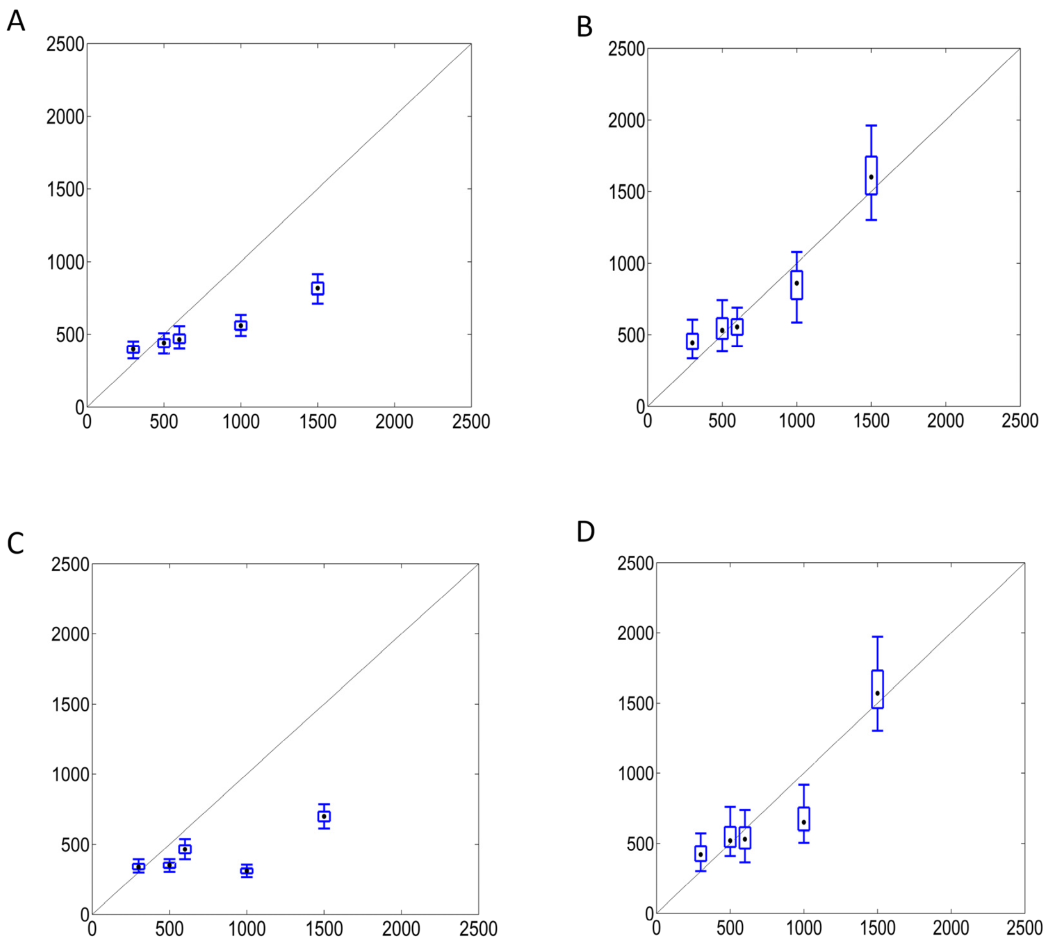

Figure S2 Box plots for effects of gene influx from population Pop8 to Pop1 and Pop4. Expected population divergence was presented on Y-axis and estimate of population divergence was shown on X-axis. The results for scenario with a gene influx of 6 per 60 haploid genomes were showed on Figure S2 A&B; the results for scenario with a gene influx of 20 per 60 haploid genomes were presented in Figure S2 C&D. The Figure S2 A&C presented results of Goldstein's method; the Figure S2 B&D presented results of the novel method.

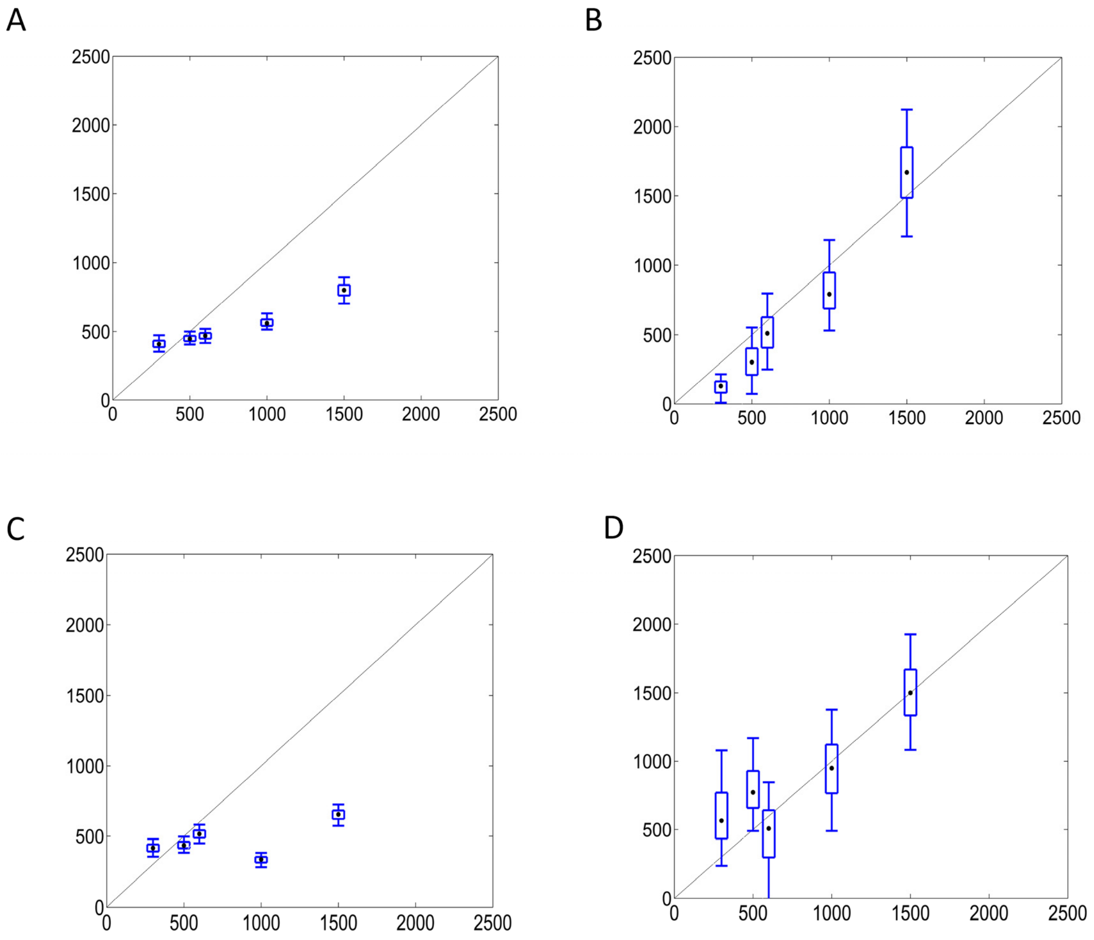

Supplement: Text S1 — Effects of genetic admixture on the novel method. (PDF) [file pone.0044788.s001.pdf]
