# Supplementary material for: Genetic Divergence Disclosing a Rapid Prehistorical Dispersion of Native Americans in Central and South America
Source: PLoS One. 2012 Sep 6;7(9):e44788. doi: 10.1371/journal.pone.0044788 (PMC3435283; doi:10.1371/journal.pone.0044788)
Supplement: Text S2 — A system of equations based on the four-population model. (PDF) [file pone.0044788.s002.pdf]

Text S2 A system of equations based on the four-population model

In the online supporting text, we showed a system of equations (Equations S1-7) based on the four-population model described in the main text (Figure 1). The equations presented relationship between expected coalescent time of pairwise lineages and demographic parameters under the assumption of exponential growth. Demographic parameters were estimated by solving the system of equations in this study. In the deduction below, the same principles were followed as that aforementioned two-population example (equation 2-4 in main text).

When both of the pairwise lineages are from population PopA (Figure 1), we have the equation below for expected coalescent time of the pairwise lineages *E(*ξ_a*),*

$$
\begin{aligned}
E(\xi_a) &= \sum_{i=1}^{\infty} i \Pr(\xi_a = i) = \sum_{i=1}^{t_{ab}} [\frac{i}{N_a(j)}] \\
&+ \sum_{i=t_{ab}+1}^{t_{abc}} \frac{i}{N_{ab}(i)} \prod_{k=1}^{t_{ab}} [1-\frac{1}{N_a(k)}] \prod_{j=t_{ab}+1}^{i-1} [1-\frac{1}{N_{ab}(j)}] \\
&+ \sum_{i=t_{abc}+1}^{t_{abcd}} \frac{i}{N_{abc}(i)} \prod_{k=1}^{t_{ab}} [1-\frac{1}{N_a(k)}] \prod_{j=t_{ab}+1}^{t_{abc}} [1-\frac{1}{N_{ab}(j)}] \prod_{m=t_{abc}+1}^{i-1} [1-\frac{1}{N_{abc}(m)}] \\
&+ \sum_{i=t_{abcd}+1}^{\infty} \frac{i}{N_{abcd}(i)} \prod_{k=1}^{t_{ab}} [1-\frac{1}{N_a(k)}] \prod_{j=t_{ab}+1}^{t_{abc}} [1-\frac{1}{N_{ab}(j)}] \prod_{m=t_{abc}+1}^{t_{abcd}} [1-\frac{1}{N_{abc}(m)}] \prod_{n=t_{abcd}+1}^{i-1} [1-\frac{1}{N_{abcd}(i)}]
\end{aligned}
\quad .
$$

Where $N_x(i)$ is the value of effective population size N_x (counting in number of effective chromosome) at generation *i* BP. And we gave

$$
\begin{aligned}
N_{ab}(i) &= N_a(i) + N_b(i) \\
N_{abc}(i) &= N_{ab}(i) + N_c(i) \\
N_{abcd}(i) &= N_{abc}(i) + N_d(i)
\end{aligned}
\quad .
$$

With the assumption that populations grow exponentially ($N^{(i)} = N^{(0)} e^{-ri}$), the $E(\xi_a)$ could be rewrite as

$$E(\xi_a)=\sum_{i=1}^{t_{ab}}\frac{ie^{ri}}{N_a}\exp[\frac{e^{ri}-e^{r}}{N_a(1-e^{r})}]$$
$$+\exp[\frac{e^{r(t_{ab}+1)}-e^{r}}{N_a(1-e^{r})}]\sum_{i=t_{ab}+1}^{t_{abc}}\frac{ie^{ri}}{N_{ab}}\exp[\frac{e^{ri}-e^{r(t_{ab}+1)}}{N_{ab}(1-e^{r})}] \qquad \text{(S1)}$$
$$+\exp[\frac{e^{r(t_{ab}+1)}-e^{r}}{N_a(1-e^{r})}]\exp[\frac{e^{r(t_{abc}+1)}-e^{r(t_{ab}+1)}}{N_{ab}(1-e^{r})}]\sum_{i=t_{abc}+1}^{t_{abcd}}\frac{ie^{ri}}{N_{abc}}\exp[\frac{e^{ri}-e^{r(t_{abc}+1)}}{N_{abc}(1-e^{r})}]$$
$$+\exp[\frac{e^{r(t_{ab}+1)}-e^{r}}{N_a(1-e^{r})}]\exp[\frac{e^{r(t_{abc}+1)}-e^{r(t_{ab}+1)}}{N_{ab}(1-e^{r})}]\exp[\frac{e^{r(t_{abcd}+1)}-e^{r(t_{abc}+1)}}{N_{abc}(1-e^{r})}]\sum_{i=t_{abcd}+1}^{\infty}\frac{ie^{ri}}{N_{abcd}}\exp[\frac{e^{ri}-e^{r(t_{abcd}+1)}}{N_{abcd}(1-e^{r})}]$$

In the same model (Figure 1), we have the expectations of expected coalescent times of the pairwise lineages ($E(\xi_b)$, $E(\xi_c)$, $E(\xi_d)$, $E(\xi_{ab})$, $E(\xi_{ac})$ and $E(\xi_{ad})$) as below,

$$E(\xi_b)=\sum_{i=1}^{t_{ab}}\frac{ie^{ri}}{N_b}\exp[\frac{e^{ri}-e^{r}}{N_b(1-e^{r})}]$$
$$+\exp[\frac{e^{r(t_{ab}+1)}-e^{r}}{N_b(1-e^{r})}]\sum_{i=t_{ab}+1}^{t_{abc}}\frac{ie^{ri}}{N_{ab}}\exp[\frac{e^{ri}-e^{r(t_{ab}+1)}}{N_{ab}(1-e^{r})}] \qquad \text{(S2)}$$
$$+\exp[\frac{e^{r(t_{ab}+1)}-e^{r}}{N_b(1-e^{r})}]\exp[\frac{e^{r(t_{abc}+1)}-e^{r(t_{ab}+1)}}{N_{ab}(1-e^{r})}]\sum_{i=t_{abc}+1}^{t_{abcd}}\frac{ie^{ri}}{N_{abc}}\exp[\frac{e^{ri}-e^{r(t_{abc}+1)}}{N_{abc}(1-e^{r})}]$$
$$+\exp[\frac{e^{r(t_{ab}+1)}-e^{r}}{N_b(1-e^{r})}]\exp[\frac{e^{r(t_{abc}+1)}-e^{r(t_{ab}+1)}}{N_{ab}(1-e^{r})}]\exp[\frac{e^{r(t_{abcd}+1)}-e^{r(t_{abc}+1)}}{N_{abc}(1-e^{r})}]\sum_{i=t_{abcd}+1}^{\infty}\frac{ie^{ri}}{N_{abcd}}\exp[\frac{e^{ri}-e^{r(t_{abcd}+1)}}{N_{abcd}(1-e^{r})}]$$

$$E(\xi_c)=\sum_{i=1}^{t_{abc}}\frac{ie^{ri}}{N_c}\exp[\frac{e^{ri}-e^{r}}{N_c(1-e^{r})}]$$
$$+\exp[\frac{e^{r(t_{abc}+1)}-e^{r}}{N_c(1-e^{r})}]\sum_{i=t_{abc}+1}^{t_{abcd}}\frac{ie^{ri}}{N_{abc}}\exp[\frac{e^{ri}-e^{r(t_{abc}+1)}}{N_{abc}(1-e^{r})}] \qquad \text{(S3)}$$
$$+\exp[\frac{e^{r(t_{abc}+1)}-e^{r}}{N_c(1-e^{r})}]\exp[\frac{e^{r(t_{abcd}+1)}-e^{r(t_{abc}+1)}}{N_{abc}(1-e^{r})}]\sum_{i=t_{abcd}+1}^{\infty}\frac{ie^{ri}}{N_{abcd}}\exp[\frac{e^{ri}-e^{r(t_{abcd}+1)}}{N_{abcd}(1-e^{r})}]$$

$$E(\xi_d)=\sum_{i=1}^{t_{abcd}}\frac{ie^{ri}}{N_d}\exp[\frac{e^{ri}-e^{r}}{N_d(1-e^{r})}] \qquad \text{(S4)}$$
$$+\exp[\frac{e^{r(t_{abcd}+1)}-e^{r}}{N_d(1-e^{r})}]\sum_{i=t_{abcd}+1}^{\infty}\frac{ie^{ri}}{N_{abcd}}\exp[\frac{e^{ri}-e^{r(t_{abcd}+1)}}{N_{abcd}(1-e^{r})}]$$

$$E(\xi_{ab})=\sum_{i=t_{ab}+1}^{\infty}i\Pr(\xi_{ab}=i)=\sum_{i=t_{ab}+1}^{t_{abc}}\frac{ie^{ri}}{N_{ab}}\exp[\frac{e^{ri}-e^{r(t_{ab}+1)}}{N_{ab}(1-e^{r})}]$$
$$+\exp[\frac{e^{r(t_{abc}+1)}-e^{r(t_{ab}+1)}}{N_{ab}(1-e^{r})}]\sum_{i=t_{abc}+1}^{t_{abcd}}\frac{ie^{ri}}{N_{abc}}\exp[\frac{e^{ri}-e^{r(t_{abc}+1)}}{N_{abc}(1-e^{r})}] \qquad \text{(S5)}$$
$$+\exp[\frac{e^{r(t_{abc}+1)}-e^{r(t_{ab}+1)}}{N_{ab}(1-e^{r})}]\exp[\frac{e^{r(t_{abcd}+1)}-e^{r(t_{abc}+1)}}{N_{abc}(1-e^{r})}]\sum_{i=t_{abcd}+1}^{\infty}\frac{ie^{ri}}{N_{abcd}}\exp[\frac{e^{ri}-e^{r(t_{abcd}+1)}}{N_{abcd}(1-e^{r})}]$$

$$E(\xi_{ac}) = \sum_{i=t_{abc}+1}^{\infty} i\,\Pr(\xi_{ac}=i) = \sum_{i=t_{abc}+1}^{t_{abcd}} \frac{ie^{ri}}{N_{abc}} \exp[\frac{e^{ri}-e^{r(t_{abc}+1)}}{N_{abc}(1-e^{r})}] + \exp[\frac{e^{r(t_{abcd}+1)}-e^{r(t_{abc}+1)}}{N_{abc}(1-e^{r})}] \sum_{i=t_{abcd}+1}^{\infty} \frac{ie^{ri}}{N_{abcd}} \exp[\frac{e^{ri}-e^{r(t_{abcd}+1)}}{N_{abcd}(1-e^{r})}] \tag{S6}$$

$$E(\xi_{ad}) = \sum_{i=t_{abcd}+1}^{\infty} i\,\Pr(\xi_{ad}=i) = \sum_{i=t_{abcd}+1}^{\infty} \frac{ie^{ri}}{N_{abcd}} \exp[\frac{e^{ri}-e^{r(t_{abcd}+1)}}{N_{abcd}(1-e^{r})}] \tag{S7}$$
